# Targeting Anhedonia in Depression: findings from a Randomized Single-Blind Comparison of arTMS and Standard rTMS

**DOI:** 10.1192/j.eurpsy.2026.11750

**Published:** 2026-07-31

**Authors:** Michele Prato, Barbara Barbini, Federico Seghi, Nicola Ragone, Carolina Passani, Cristina Colombo

**Affiliations:** 1 Vita-Salute San Raffaele University, Department of Clinical Neurosciences, Milan, Italy; 2 Department of Psychiatry, Mood Disorder Unit, Scientific Institute IRCCS San Raffaele Hospital, Milan, Italy

## Abstract

**Introduction:**

Anhedonia, defined as the diminished ability to experience pleasure, is a core symptom of Depression and is associated with significant functional impairment, increased suicidality, and poor treatment outcomes (Rizvi et al., Neurosci Biobehav Rev 2016;65:21-35). This symptom often persists even after mood symptoms remit, suggesting that anhedonia may represent a distinct therapeutic target with unique clinical and neurobiological characteristics (Cooper et al., Front Psychiatry 2018;9:513). For this reason, it has more recently become a focus of novel therapeutic strategies, including repetitive Transcranial Magnetic Stimulation (rTMS), which has shown preliminary efficacy in modulating reward-related symptoms **(Fukuda et al., Brain Behav 2021;11(9):e2329)**.

**Objectives:**

This study compared the effects of accelerated repetitive Transcranial Magnetic Stimulation (arTMS) versus standard rTMS on anhedonia in inpatients with Major Depressive Episodes (MDEs), including both unipolar and bipolar depression. Accelerated rTMS, which delivers multiple sessions per day over a shorter period, reduces treatment duration and is emerging as an effective alternative to standard rTMS.

**Methods:**

In a single-blind randomized design, 33 inpatients diagnosed with MDE were assigned to receive either arTMS (4 sessions/day for 5 days; n=18) or standard rTMS (1 session/day for 4 weeks; n=15). Due to 2 dropouts in the standard rTMS group, the final analysis included 18 patients in the arTMS group and 13 in the rTMS group. Anhedonia was assessed using Item 8 of the Montgomery–Åsberg Depression Rating Scale (MADRS) and Items 4 and 12 of the Beck Depression Inventory-II (BDI-II) at baseline, day 28, and day 56.

**Results:**

At day 28, MADRS Item 8 scores decreased by 2.1 points in the arTMS group compared to 0.8 in the rTMS group (p=0.003); at day 56, reductions were 2.4 and 1.0, respectively (p=0.002). For BDI-II Item 4, reductions at day 56 were 1.3 in the arTMS group versus 0.7 in the rTMS group (p=0.008); for Item 12, 1.0 versus 0.4 (p=0.01). No significant differences in adverse events were observed between groups.

**Conclusions:**

These preliminary findings suggest that arTMS may lead to faster and greater improvements in anhedonia symptoms compared to standard rTMS in inpatients with MDE, without compromising safety or tolerability. This supports the potential of arTMS as a promising intervention for persistent symptoms like anhedonia, which often remain refractory to conventional treatments. Further large-scale, controlled studies are needed to confirm these results and clarify the underlying neurobiological mechanisms, including modulation of reward and motivational neural networks.References:

1. Rizvi SJ, Pizzagalli DA, Sproule BA, Kennedy SH. Assessing anhedonia in depression: Potentials and pitfalls. *Neurosci Biobehav Rev.* 2016;65:21–35. doi:10.1016/j.neubiorev.2016.03.004.
2. Cooper JA, Arulpragasam AR, Treadway MT. Anhedonia in depression: biological mechanisms and computational models. *Curr Opin Behav Sci.* 2018;22:128–135. doi:10.1016/j.cobeha.2018.01.024.
3. Fukuda AM, Kang JWD, Gobin AP, Tirrell E, Kokdere F, Carpenter LL. Effects of transcranial magnetic stimulation on anhedonia in treatment resistant major depressive disorder. *Brain Behav.* 2021;11(9):e2329. doi:10.1002/brb3.2329.

